# Forward- and Inverse-Planned Intensity-Modulated Radiotherapy in the CHHiP Trial: A Comparison of Dosimetry and Normal Tissue Toxicity

**DOI:** 10.1016/j.clon.2019.05.002

**Published:** 2019-09

**Authors:** O.F. Naismith, C. Griffin, I. Syndikus, C. South, H. Mayles, P. Mayles, V. Khoo, C. Scrase, J. Graham, S. Hassan, E. Hall, D.P. Dearnaley, the CHHiP Investigators

**Affiliations:** ∗ Royal Marsden NHS Foundation Trust, London, UK; † The Institute of Cancer Research, London, UK; ‡ Clatterbridge Cancer Centre, Wirral, UK; § Royal Surrey County Hospital, Guildford, UK; ¶ Ipswich Hospital, Ipswich, UK; || Beacon Centre, Musgrove Park Hospital, Taunton, UK

**Keywords:** DVH, forward planning, intensity-modulated radiotherapy, inverse planning, prostate cancer, toxicity

## Abstract

**Aims:**

The CHHiP (Conventional or Hypofractionated High-dose Intensity Modulated Radiotherapy In Prostate Cancer; CRUK/06/016) trial investigated hypofractionated radiotherapy for localised prostate cancer. Forward- (FP) or inverse-planned (IP) intensity-modulated techniques were permitted. Dose–volume histogram and toxicity data were compared to explore the effects of planning method.

**Materials and methods:**

In total, 337 participants with intermediate-risk disease and prospectively collected toxicity data were included. Patients were matched on prostate and rectum/bladder volumes and on radiotherapy dose for toxicity comparisons. The primary outcome was grade 2 or higher Radiation Therapy Oncology Group (RTOG) bowel or bladder toxicity at 2 years.

**Results:**

IP patients had smaller volumes of rectum irradiated to 50–70 Gy (*P* < 0.001); FP patients had smaller volumes of bladder irradiated to 74 Gy (*P* = 0.001). Acute grade 2 + bowel toxicity was worse with FP (27/53 [52%]; 11/53 [21%] IP; *P* = 0.0002); with no significant differences in acute urinary toxicity. At 2 years, RTOG grade 2 + bowel toxicity rates were FP 0/53 and IP 2/53 and RTOG grade 2 + bladder rates were FP 0/54 and IP 1/57.

**Conclusions:**

Significant differences were found between dose–volume histograms from FP and IP methods. IP may result in small reductions in acute bowel toxicity but both techniques were associated with low rates of late radiotherapy side-effects.

## Highlights

• Inverse planning resulted in smaller volumes of the rectum irradiated to 50–70 Gy.
• Inverse planning resulted in larger volumes of the bladder irradiated to 74 Gy.
• Inverse-planned patients had less acute bowel toxicity.
• Late toxicity was low for forward and inverse planning.

## Introduction

Prostate cancer is the most common cancer among men in the developed world [1], [2] and radiotherapy is a curative treatment option. The conventional radiotherapy dose is limited by both acute and late side-effects in organs at risk (OAR) located in close proximity to the target volume; conformal radiotherapy (3DCRT) gave the opportunity for dose escalation [3]. There is a clear relationship between increasing radiation dose and improved clinical outcome (biochemical progression-free survival) [4]. Intensity-modulated radiotherapy (IMRT) has proven to be a powerful technique in terms of its dosimetric benefits for complex treatment sites and has become widely adopted for the treatment of prostate cancer [5], [6], [7], [8].

CHHiP (Conventional or Hypofractionated High-dose Intensity Modulated Radiotherapy In Prostate Cancer; CRUK/06/016) was a randomised phase III trial in men with localised prostate cancer. It showed that hypofractionated radiotherapy (60 Gy/20 fractions) is safe and non-inferior to conventionally fractionated (74 Gy/37 fractions) in terms of the time to biochemical/clinical failure [9]. Radiotherapy treatment within CHHiP used a complex target volume treated with IMRT. When the trial was initiated in 2002, IMRT was a relatively new technique in the UK and was unavailable or restricted in clinical application at many centres [7]. Hence, both forward-planned (FP) and inverse-planned (IP) IMRT were permitted. The FP technique used a multi-segment three-field plan with optimal beam angles which had been compared with the two-phase 3DCRT technique used in the previous Medical Research Council (MRC) RT01 trial [4], [10]. All CHHiP FP plans produced lower mean irradiated rectal volumes at all measured dose levels compared with the RT01 plans and also gave lower mean irradiated bladder volumes at both 50 and 60 Gy.

The study reported here compares dose–volume histogram (DVH) and rectum and bladder toxicity data for patients planned and treated using FP and IP techniques in the CHHiP trial. The aim was to determine if there were any systematic differences resulting from the two planning techniques. The analyses were planned and conducted in two stages. The first stage analysed dosimetry data to determine the relative merits of FP and IP on rectal and bladder DVHs; the second stage investigated whether any differences in the DVH data translated into clinically observable benefits in terms of a reduction in side-effects.

## Materials and Methods

### Trial Design

Full details of the CHHiP trial design, eligibility and treatment have been previously reported [9]. Briefly, men with histologically confirmed T1b-T3a,N0,M0 prostate cancer [11] suitable for radiotherapy were eligible. Patients were randomised (in a 1:1:1 ratio) to conventional fractionation (74 Gy/37 fractions over 7.4 weeks) or one of two hypofractionated schedules (60 Gy/20 fractions/4.0 weeks or 57 Gy/19 fractions/3.8 weeks). Randomisation was stratified by National Comprehensive Cancer Network (NCCN) risk classification (low versus intermediate versus high) [12] and radiotherapy treatment centre. Treatment allocation was not masked.

### Treatment Details

Target volumes and doses are summarised in Supplementary Figure S1, with the core high-dose region receiving the target dose of 74, 60 or 57 Gy in accordance with allocated treatment [13].

Patients were computed tomography scanned at ≤5 mm intervals with a comfortably full bladder and an empty rectum, using approved immobilisation methods. Radiotherapy treatment used either a single-phase FP method (field-in-field or segmented-field arrangement with three beam angles) or five-field IP IMRT, with ‘step and shoot’ or ‘dynamic leaf’ delivery. (Rotational arc delivery was permitted but was not widely used at the time of the trial.)

OARs included the bladder, rectum, bowel and femoral heads. The entire bladder was outlined. The outer wall of the rectum was outlined from the anus (at the level of the ischial tuberosities or 1 cm below the lower margin of the planning target volume [PTV], whichever was more inferior) to the recto-sigmoid junction. OAR dose constraints were applied for treatment plan optimisation, defined for the conventional fractionation arm and linearly scaled to the same percentage of prescribed dose for the hypofractionated schedules. The rectum dose constraints were V74Gy < 3%, V70Gy < 15%, V65Gy < 30%, V60Gy < 50% and V50Gy < 60%. The bladder dose constraints were V74Gy < 5%, V60Gy < 25% and V50Gy < 50%. A plan assessment form, which provided a synopsis of DVH data for PTVs and OARs, was completed by the treatment centre for each patient treatment plan.

The planning methods, treatment delivery and verification techniques used within each centre were identical for each fractionation regimen and were reviewed and approved in advance by the national Radiotherapy Trials Quality Assurance group. Within a centre, different planning techniques were permitted for low- and intermediate-/high-risk groups.

### Patients

**Fig 1:**
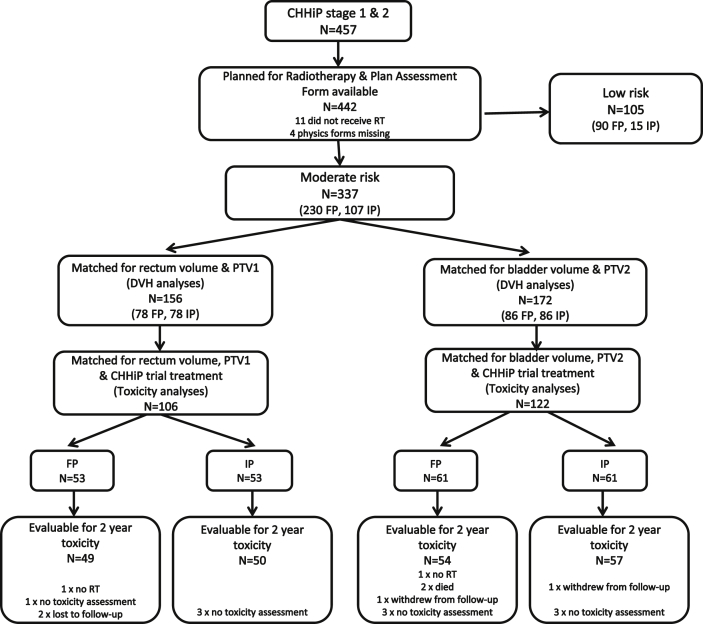
CONSORT flow diagram for CHHiP forward-planned (FP) and inverse-planned (IP) analyses.

CHHiP was conducted in three stages; this report uses toxicity data from stages 1 and 2 (safety), which between 18 October 2002 and 12 August 2006 recruited 457 patients from 11 UK centres using six different treatment planning systems [13]. Radiotherapy planning data were available for 442/457 patients; 337 had intermediate-risk disease and 105 had low-risk disease. The 105 low-risk patients were excluded from all analyses due to the small numbers of IP patients (15/105) (Figure 1).

### Statistical Considerations

#### Volume-Matching Procedure

To reduce the potential for bias in the non-randomised comparisons of planning technique, analysis sets balanced for key variables that might affect the relationship between planning method and radiation dose to the bladder/rectum were defined. In particular, the centre treating the majority of IP patients was unique in using daily rectal micro-enemas, and there was significant variability between centres in the drinking volumes recommended by their bladder preparation procedures (200–750 ml). PTV differences resulting from the margin-growing algorithms of the various treatment planning systems have also been reported [14]. IP and FP patients were matched (1:1) using two volume parameters, each divided into six volume bands. For rectum DVH analyses, patients were matched according to PTV1 volume (including seminal vesicles) and rectal volume; for bladder DVH analyses, PTV2 and bladder volumes were used for matching.

#### Dose–Volume Histogram Comparison

Dose–volume data recorded on the plan assessment form for the rectum and bladder were compared. Although the three trial treatment groups differed in prescription dose and fractionation, the dose constraints, when scaled as a percentage of the prescribed dose, were identical, and each treatment group was planned and normalised in the same way within a treatment centre. DVH data could thus be compared directly in this planning study using relative dose without regard for treatment group. Descriptive statistics and boxplots were used to summarise the DVH data. The Mann–Whitney test was used to compare the distribution of data at each dose level between planning methods.

#### Toxicity Comparison

The second stage was to investigate whether any observed differences in normal tissue dosimetry were associated with normal tissue toxicity. Dose and fractionation could potentially bias these results and so, for toxicity analysis, patients were additionally matched according to treatment dose schedule.

Acute side-effects were assessed using the Radiation Therapy Oncology Group (RTOG) scoring system for acute toxicity [15], completed weekly during treatment and at weeks 10, 12 and 18 from the radiotherapy start date. Late side-effects were assessed at 6, 12, 18 and 24 months using RTOG, the Late Effects on Normal Tissues: Subjective: Objective/Management (LENT/SOM) and Royal Marsden Hospital (RMH) scoring systems [16], [17], [18]. Patient-reported outcomes (PRO) were assessed before trial entry, before radiotherapy and at week 10, and 6, 12, 18 and 24 months after radiotherapy using the UCLA Prostate Cancer Index questionnaire [19]. The primary toxicity end point for this analysis was grade 2 or greater RTOG bladder or bowel toxicity experienced 2 years from the start of radiotherapy.

Baseline characteristics were summarised using descriptive statistics and, as the two groups were not generated by random allocation, statistical comparisons were made between the groups using chi-squared or Mann–Whitney tests as appropriate. Patients were only included in toxicity analyses if they received at least one fraction of radiotherapy.

Toxicity and PRO data are presented as grade distributions at each time point and compared using Mann–Whitney tests. The proportion of patients with grade 2 + RTOG bladder or bowel toxicity at 2 years is presented together with exact binomial confidence intervals. The time from the radiotherapy start date to the first occurrence of grade 1 + toxicity was analysed using Kaplan–Meier methods used to estimate the cumulative proportion with an event at 2 years. All data reported were used; patients with no event were censored on the date of their last toxicity assessment. Cox proportional hazard models were used to estimate and test the effect of planning method (using the Wald test) with a hazard ratio of less than 1 favouring IP. The proportional hazards assumption was found to hold for all time-to-event analyses reported. Changes in PRO scores between the pre-radiotherapy assessment and after 2 years were calculated and are presented graphically.

All analyses were exploratory in nature. However, statistical analysis plans were written before conducting each of the pre-planned stages. A significance level of 1% was used to allow for multiple testing. Analyses were based on a database snapshot taken on 1 April 2010 and were conducted using Stata version 11.2.

## Results

### Volume Matching

There was considerable imbalance between the rectum, bladder, PTV1 and PTV2 volumes in the FP and IP groups, with larger volumes for all four structures in the FP group. Following the volume-matching process there were no significant differences between FP and IP groups (*P* > 0.05 for rectum, bladder, PTV1 and PTV2) (Supplementary Figure S2). Seventy-eight FP–IP pairs of patients were matched on rectum and PTV1 volume (i.e. 156/337 available patient datasets) and 86 pairs matched on bladder and PTV2 volume (172/337). Following additional matching on trial treatment allocation, the number of pairs for toxicity analyses was reduced further to 53 for the rectum dataset (106/337) and 61 for the bladder (122/337) (Supplementary Table S1). There was reasonable balance in the clinical baseline characteristics of the matched datasets (Supplementary Table S2). Initial prostate-specific antigen levels were lower in the IP group for the bladder but not the rectum subset. More patients in the FP group required a modification to the posterior target volume margins in both rectum (FP 4 [8%]: IP 0 [0%]) and bladder (FP 6 [10%]: IP 1 [2%]) subsets. Derivation of the patient datasets for analysis is summarised in Figure 1.

### Dose–Volume Histogram Analysis

**Fig 2:**
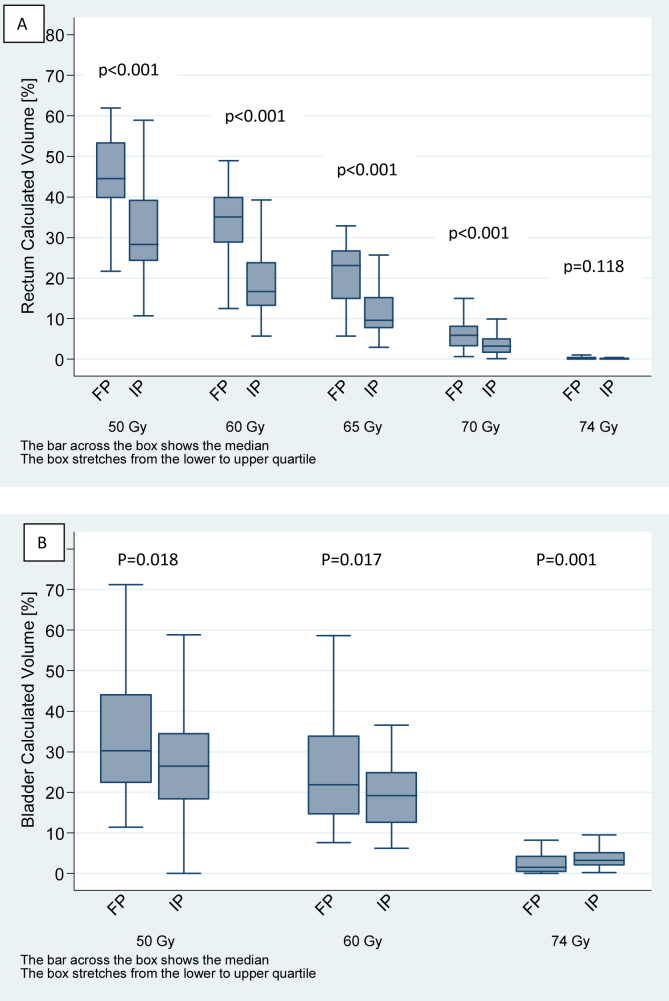
Boxplots illustrating the distribution of (A) rectum volumes and (B) bladder volumes for forward-planned (FP) and inverse-planned (IP) groups for each dose constraint.

For the rectum (Figure 2A), IP patients had significantly smaller volumes of their rectum irradiated to doses of 50 Gy (median: 43.7% FP, 27.2% IP), 60 Gy (median: 34.3% FP, 16.0% IP), 65 Gy (median: 22.1% FP, 9.5% IP) and 70 Gy (median: 6.3% FP, 2.9% IP) compared with FP patients (*P* < 0.001). No difference was apparent at 74 Gy due to the small volumes receiving this dose. By contrast, IP patients had significantly larger volumes of bladder irradiated to 74 Gy (median 1.7% FP, 3.2% IP) than FP patients (*P* = 0.001) (Figure 2B). Differences between bladder volumes irradiated to 50 and 60 Gy were not statistically significant, but IP tended to result in lower bladder DVH volumes at these doses.

### Toxicity

**Fig 3:**
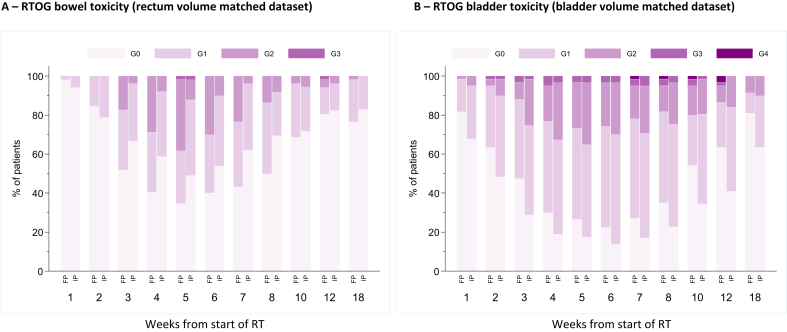
Distribution of acute Radiation Therapy Oncology Group (RTOG) toxicity by planning method: (A) bowel toxicity (in rectum volume matched dataset) and (B) bladder toxicity (in bladder volume matched dataset).

**Fig 4:**
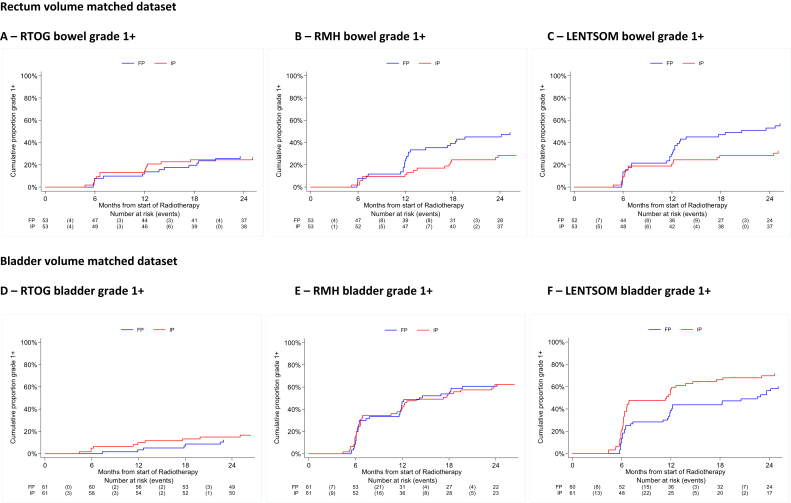
Cumulative proportion of grade 1 + toxicity assessed by Radiation Therapy Oncology Group (RTOG), Royal Marsden Hospital (RMH) and Late Effects on Normal Tissues: Subjective: Objective/Management (LENT/SOM) scoring systems. (A–C) Bowel toxicity for rectum volume matched dataset and (D–F) bladder toxicity for bladder volume matched dataset.

There was a statistically significant difference in the worst acute bowel toxicity (*P* = 0.0002), with 52% (27/52) FP compared with 21% (11/53) IP experiencing grade 2 + toxicity during the first 18 weeks from the start of radiotherapy (Figure 3A). Late toxicity was low with both planning methods, with 0/49 (0%; 95% confidence interval 0–7.2%) and 1/50 (2.0%; 95% confidence interval 0.1–10.6%) RTOG bowel grade 2 + events in the FP and IP groups, respectively, at 2 years (Table 1). The RMH and LENT/SOM tools suggested benefits for IP at almost all time points from 6 to 24 months (Table 1), although the only statistically significant difference was for LENT/SOM assessment at 18 months (*P* = 0.008). The time to first post-radiotherapy grade 1 + RMH and LENT/SOM bowel toxicity was reduced for FP patients compared with IP patients (RMH grade 1+: hazard ratio = 0.40; 95% confidence interval 0.21–0.73; *P* = 0.003; LENT/SOM grade 1+: hazard ratio = 0.48; 95% confidence interval 0.27–0.84; *P* = 0.01), although this was not seen with RTOG assessment (Figure 4 and Supplementary Table S3). However, there was no difference for grade 2 + or 3 + events using any scoring system (Supplementary Table S3), but the number of events was very small. PROs showed an approximate doubling of ‘overall bowel problems’, ‘distress’ and ‘rectal urgency’ at week 10, in keeping with the physician-based scores. However, no consistent differences in PROs between planning methods remained from months 6–24 when outcomes appeared very favourable in both groups (Table 2). Change scores from pre-radiotherapy to 24 months confirmed the generally favourable bowel outcomes and similarities between planning methods (Supplementary Figure S3).

**Table 1:** Late toxicity assessed using Radiation Therapy Oncology Group (RTOG), Royal Marsden Hospital (RMH) and Late Effects on Normal Tissues: Subjective: Objective/Management (LENT/SOM) scoring systems: bowel toxicity (in rectum volume matched dataset) and bladder toxicity (in bladder volume matched dataset)

Grade		Rectum volume matched dataset						Bladder volume matched dataset					
		RTOG bowel		RMH bowel		LENT/SOM bowel		RTOG bladder		RMH bladder		LENT/SOM bladder	
		FP	IP	FP	IP	FP	IP	FP	IP	FP	IP	FP	IP
		n = 53	n = 53	n = 53	n = 53	n = 53	n = 53	n = 61	n = 61	n = 61	n = 61	n = 61	n = 61
		n (%)	n (%)	n (%)	n (%)	n (%)	n (%)	n (%)	n (%)	n (%)	n (%)	n (%)	n (%)
6 months	0	44 (90)	46 (87)	43 (88)	47 (90)	38 (78)	43 (81)	59 (98)	57 (93)	40 (67)	40 (66)	43 (72)	31 (51)
	1	3 (6)	7 (13)	4 (8)	5 (10)	9 (18)	7 (13)	0	4 (7)	16 (27)	19 (31)	10 (17)	21 (34)
	2	2 (4)	0	2 (4)	0	1 (2)	2 (4)	1 (2)	0	3 (5)	2 (3)	6 (10)	8 (13)
	3	0	0	0	0	1 (2)	1 (2)	0	0	1 (2)	0	1 (2)	1 (2)
	4	0	0	0	0	0	0	0	0	0	0	0	0
	Not assessed	4	0	4	1	4	0	1	0	1	0	1	0
Mann–Whitney P value		0.701		0.628		0.699		0.188		0.985		0.036	
12 months	0	44 (88)	45 (85)	35 (70)	46 (87)	33 (67)	40 (76)	56 (97)	58 (95)	36 (62)	41 (67)	42 (72)	29 (48)
	1	4 (8)	7 (13)	13 (26)	6 (9)	13 (27)	11 (21)	1 (2)	2 (3)	18 (31)	17 (28)	10 (17)	25 (41)
	2	2 (4)	1 (2)	1 (2)	1 (2)	2 (4)	2 (4)	1 (2)	1 (2)	1 (2)	2 (3)	4 (7)	6 (10)
	3	0	0	1 (2)	1 (2)	1 (2)	0	0	0	3 (5)	1 (2)	2 (4)	1 (2)
	4	0	0	0	0	0	0	0	0	0	0	0	0
	Not assessed	3	0	3	0	4	0	3	0	3	0	3	0
Mann–Whitney P value		0.690		0.047		0.350		0.697		0.524		0.016	
18 months	0	39 (81)	49 (94)	35 (73)	46 (89)	32 (67)	46 (89)	50 (93)	56 (93)	34 (62)	38 (63)	38 (72)	35 (59)
	1	7 (15)	2 (4)	11 (23)	5 (10)	10 (21)	4 (8)	3 (6)	3 (5)	19 (35)	20 (33)	4 (8)	18 (31)
	2	2 (4)	1 (2)	1 (2)	1 (2)	4 (8)	2 (4)	1 (2)	0	1 (2)	1 (2)	9 (17)	5 (9)
	3	0	0	1 (2)	0	2 (4)	0	0	1 (2)	1 (2)	0	2 (4)	0
	4	0	0	0	0	0	0	0	0	0	1 (2)	0	1 (2)
	Not assessed	5	1	5	1	5	1	7	1	6	1	8	2
Mann–Whitney P value		0.049		0.050		0.008		0.883		0.866		0.460	
24 months	0	44 (90)	48 (96)	33 (67)	44 (88)	32 (65)	42 (86)	51 (94)	56 (98)	37 (69)	39 (68)	34 (63)	34 (60)
	1	5 (10)	1 (2)	15 (31)	5 (10)	14 (19)	6 (12)	3 (6)	0	14 (26)	14 (25)	10 (19)	17 (30)
	2	0	1 (2)	1 (2)	1 (2)	2 (4)	1 (2)	0	1 (2)	2 (4)	3 (5)	9 (17)	4 (7)
	3	0	0	0	0	1 (2)	0	0	0	1 (2)	1 (2)	1 (2)	2 (4)
	4	0	0	0	0	0	0	0	0	0	0	0	0
	Not assessed	4	3	4	3	4	4	7	4	5	4	7	4
Mann–Whitney P value		0.247		0.016		0.019		0.298		0.954		0.984	

FP, forward planned; IP, inverse planned.

**Table 2:** Distribution of bowel and urinary symptoms from the UCLA Prostate Cancer Index questionnaire before radiotherapy and at week 10, and months 6, 12, 18 and 24 from the start of radiotherapy. Bowel symptoms are presented in the rectum volume matched dataset and urinary symptoms in the bladder volume matched dataset

Bowel habits (rectum volume matched dataset)	Before radiotherapy		Week 10		6 months		12 months		18 months		24 months	
	FP	IP	FP	IP	FP	IP	FP	IP	FP	IP	FP	IP
	n = 42	n = 42	n = 31	n = 30	n = 40	n = 42	n = 40	n = 40	n = 40	n = 37	n = 40	n = 38
Frequency of rectal urgency												
More than once a day	0	2 (5)	6 (19)	1 (3)	4 (10)	2 (5)	0	2 (5)	5 (13)	1 (3)	1 (2)	1 (3)
Once a day	6 (14)	3 (7)	4 (13)	4 (13)	1 (3)	4 (10)	4 (10)	5 (13)	3 (7)	1 (3)	1 (2)	1 (3)
More than once a week	0	1 (2)	3 (10)	2 (7)	5 (12)	5 (12)	0	2 (5)	1 (3)	0	2 (5)	2 (5)
Once a week	2 (5)	2 (5)	3 (10)	6 (20)	3 (7)	6 (14)	4 (10)	1 (3)	2 (5)	5 (14)	3 (8)	5 (13)
Rarely or never	34 (81)	34 (81)	15 (48)	17 (57)	27 (68)	25 (60)	32 (80)	30 (75)	29 (73)	30 (81)	33 (83)	29 (76)
Frequency loose/liquid stools												
Never	53 (55)	20 (48)	13 (42)	9 (30)	15 (37)	7 (17)	16 (39)	8 (20)	14 (35)	13 (35)	22 (55)	16 (42)
Rarely	14 (33)	17 (41)	10 (32)	10 (33)	18 (44)	27 (64)	20 (49)	21 (53)	16 (40)	17 (46)	13 (33)	11 (29)
About half the time	4 (10)	3 (7)	7 (23)	9 (30)	5 (12)	4 (10)	2 (5)	8 (20)	6 (15)	4 (11)	4 (10)	6 (16)
Usually	1 (2)	1 (2)	1 (3)	2 (7)	2 (5)	4 (10)	2 (5)	2 (5)	3 (8)	3 (8)	1 (2)	4 (11)
Always	0	1 (2)	0	0	1 (2)	0	1 (2)	1 (3)	1 (3)	0	0	1 (3)
Distress from bowel movements												
Severe	1 (2)	0	2 (7)	1 (3)	1 (2)	0	1 (2)	1 (3)	0	0	0	0
Moderate	0	3 (7)	4 (13)	2 (7)	1 (2)	4 (10)	3 (7)	1 (3)	2 (5)	1 (3)	2 (5)	1 (3)
A little	5 (12)	9 (21)	9 (29)	13 (43)	10 (24)	10 (24)	5 (12)	8 (20)	10 (25)	6 (16)	5 (13)	7 (18)
No distress	36 (86)	30 (71)	16 (52)	14 (47)	29 (71)	28 (67)	32 (78)	30 (75)	28 (70)	30 (81)	33 (83)	30 (79)
Crampy pain												
Several times a day	0	0	2 (6)	1 (3)	2 (5)	1 (2)	0	2 (5)	1 (2)	0	0	0
Once a day	1 (2)	0	1 (3)	0	0	1 (2)	1 (2)	0	2 (5)	0	1 (2)	0
Several times a week	1 (2)	2 (5)	1 (3)	1 (3)	1 (2)	0	1 (2)	2 (5)	1 (2)	3 (8)	1 (2)	2 (5)
Once a week	5 (12)	2 (5)	1 (3)	1 (3)	1 (2)	2 (5)	0	3 (8)	0	1 (3)	1 (2)	2 (5)
Once this month	1 (2)	4 (10)	1 (3)	3 (10)	3 (7)	9 (21)	3 (7)	5 (13)	1 (2)	2 (5)	4 (10)	1 (3)
Rarely or never	33 (81)	34 (81)	25 (81)	23 (79)	34 (83)	29 (69)	36 (88)	28 (70)	35 (88)	31 (84)	33 (83)	33 (87)
Overall problem of bowel habits												
Big	0	1 (2)	1 (3)	1 (3)	1 (2)	0	1 (2)	1 (3)	0	0	0	0
Moderate	1 (2)	1 (2)	4 (13)	1 (3)	1 (2)	3 (7)	3 (7)	3 (8)	2 (5)	1 (3)	1 (2)	0
Small	3 (7)	2 (5)	4 (13)	3 (10)	3 (7)	2 (5)	0	4 (10)	5 (13)	3 (8)	1 (2)	4 (11)
Very small	3 (7)	12 (29)	8 (26)	10 (33)	11 (27)	12 (29)	8 (20)	8 (20)	7 (18)	6 (16)	12 (30)	6 (16)
No problem	35 (83)	26 (62)	14 (45)	15 (50)	25 (61)	25 (60)	29 (71)	24 (60)	26 (65)	27 (73)	26 (65)	28 (74)
Urinary function (bladder volume matched dataset)	n = 45	n = 52	n = 31	n = 35	n = 43	n = 42	n = 44	n = 49	n = 41	n = 44	n = 42	n = 41
Frequency of leaking urine												
Everyday	2 (4)	3 (6)	1 (3)	1 (3)	0	2 (5)	2 (5)	5 (10)	1 (2)	2 (5)	0	1 (2)
About once a week	2 (4)	4 (8)	2 (6)	2 (6)	3 (7)	5 (12)	2 (5)	3 (6)	5 (12)	3 (7)	7 (17)	4 (10)
Less than once a week	3 (7)	7 (13)	3 (10)	9 (26)	3 (7)	9 (21)	2 (5)	7 (14)	5 (12)	9 (21)	2 (5)	4 (10)
Not at all	38 (84)	38 (73)	25 (81)	23 (66)	37 (86)	26 (62)	38 (86)	34 (69)	30 (73)	30 (68)	33 (79)	31 (78)
Urinary control												
No control whatsoever	0	1 (2)	0	0	1 (2)	0	0	0	0	0	0	0
Frequent dribbling	1 (2)	1 (2)	0	0	0	1 (2)	0	1 (2)	0	0	0	0
Occasional dribbling	8 (18)	16 (31)	7 (23)	10 (30)	6 (14)	13 (31)	5 (11)	13 (27)	10 (24)	14 (32)	10 (24)	12 (30)
Total control	36 (80)	34 (65)	24 (78)	23 (70)	36 (84)	28 (67)	39 (89)	35 (71)	31 (76)	30 (68)	32 (76)	28 (70)
Pads or diapers required per day												
3 or more pads	0	0	0	0	0	0	0	0	0	1 (2)	0	0
1–2 pads	2 (5)	0	1 (3)	0	0	1 (2)	0	2 (4)	0	0	1 (2)	0
No pads	42 (95)	52 (100)	30 (97)	33 (100)	40 (100)	42 (98)	43 (100)	47 (96)	41 (100)	43 (98)	41 (98)	41 (100)
Dripping urine/wetting pants												
No problem	40 (91)	39 (75)	22 (71)	25 (74)	34 (79)	30 (70)	33 (79)	35 (71)	33 (81)	36 (82)	33 (79)	33 (81)
Very small problem	2 (5)	8 (15)	6 (19)	7 (21)	7 (16)	10 (23)	7 (17)	9 (18)	4 (10)	5 (11)	8 (19)	7 (17)
Small problem	1 (2)	2 (4)	2 (6)	1 (3)	2 (5)	1 (2)	2 (5)	4 (8)	3 (7)	3 (7)	0	1 (2)
Moderate problem	1 (2)	0	1 (3)	1 (3)	0	1 (2)	0	1 (2)	1 (2)	0	1 (2)	0
Big problem	0	3 (6)	0	0	0	1 (2)	0	0	0	0	0	0
Urine leakage interfering with sexual activity												
No problem	39 (93)	41 (85)	26 (93)	28 (90)	38 (93)	35 (92)	41 (98)	35 (83)	38 (97)	38 (95)	37 (88)	34 (94)
Very small problem	0	2 (4)	1 (4)	3 (10)	1 (2)	0	0	4 (10)	0	0	3 (7)	1 (3)
Small problem	2 (5)	1 (2)	0	0	2 (5)	0	0	1 (2)	0	1 (2)	0	1 (3)
Moderate problem	0	0	1 (4)	0	0	1 (3)	0	2 (5)	0	1 (2)	2 (5)	0
Big problem	1 (2)	4 (8)	0	0	0	2 (5)	1 (2)	0	1 (3)	0	0	0
Overall problem with urinary function												
No problem	30 (67)	23 (44)	10 (32)	15 (43)	28 (65)	26 (61)	33 (75)	29 (59)	29 (71)	31 (70)	31 (74)	29 (71)
Very small problem	7 (16)	15 (29)	11 (36)	11 (31)	11 (26)	13 (30)	8 (18)	12 (25)	7 (17)	8 (18)	6 (14)	8 (20)
Small problem	2 (4)	6 (12)	4 (13)	6 (17)	3 (7)	2 (5)	2 (5)	6 (12)	4 (10)	4 (9)	5 (12)	2 (5)
Moderate problem	4 (9)	6 (12)	6 (19)	2 (6)	0	2 (5)	1 (2)	2 (4)	1 (2)	1 (2)	0	2 (5)
Big problem	2 (4)	2 (4)	0	1 (3)	1 (2)	0	0	0	0	0	0	0

FP, forward planned; IP, inverse planned.

For the bladder dataset, there was no evidence of a difference in the worst acute bladder toxicity (*P* = 0.709), with 45% (27/60) FP and 46% (28/61) IP patients experiencing grade 2 + toxicity during the first 18 weeks (Figure 3B). However, grade 1 + toxicity was higher at all timepoints from weeks 1–18 in the IP group. There was no evidence of a difference for grade 2 + late bladder toxicity (Table 1), which was low in both groups. At 2 years, RTOG grade 2 + bladder toxicity was reported in 0/54 (0%; 95% confidence interval 0–6.6%) and 1/57 (1.8%; 95% confidence interval 0.1–9.4%) patients in the FP and IP groups, respectively. Time-to-event analyses indicated no statistically significant differences in bladder toxicity but there was a trend for higher grade 1 + in the IP group for RTOG and LENT/SOM scales (Figure 4 and Supplementary Table S3). Patient-reported urinary outcomes seemed to be slightly higher at baseline in the IP group. At 2 years, both groups had similarly favourable profiles (Table 2). Change scores indicated that at 24 months an improvement in overall urinary function was evident for some patients in both planning method groups (Supplementary Figure S3).

## Discussion

Careful matching of patients on rectum and bladder volumes was necessary because patients were not randomised to planning method and there were systematic differences in patient preparation techniques between the centre recruiting the majority of the IP patients and elsewhere (e.g. daily use of a rectal enema). The procedural differences resulted in a significant difference in rectum and bladder volumes, which were both smaller in the IP group, and these were successfully accounted for by the matching process.

Both FP and IP techniques were successful in achieving the rectal and bladder dose constraints. The use of IP IMRT enabled the dose to be conformed more optimally to the shape of the PTV, in particular to the concavity formed by the seminal vesicles wrapping around the rectum. This largely explains the differences seen in the IP and FP dose–volume data for the rectum, where IP reduced the volume of rectum irradiated to doses of 50 Gy and above. Both techniques were successful at limiting the rectal volume receiving the prescribed 74 Gy dose, where the PTV excluded seminal vesicles, so no difference was apparent at this dose. These results are similar to those reported in previous studies [10], [20], [21], [22], [23], where IMRT significantly reduced volumes of rectum exposed to doses >60 Gy, with no significant difference near the prescription dose.

The higher volume of bladder irradiated to 74 Gy with IP may be due to the five-field beam geometry used, which resulted in an anterior peak in the dose distribution above the PTV and up into the bladder from the overlapping of the two anterior-oblique beams. This did not occur with FP, as an orthogonal beam arrangement was used (anterior and two lateral beams) so, although the isodoses did not conform so well to the circular shape of the prostate PTVs when viewed on axial computed tomography images, the anterior shape of the isodoses was generally flat across the top of the PTV for FP. By contrast, past studies have reported a slight reduction in bladder volumes exposed to high doses with IMRT, with volumes exposed to intermediate and low doses often higher for IMRT. This may be due in part to the different beam configurations used in these studies for FP, with three to nine coplanar beams, and the use of multiphase plans instead of field-in-field techniques. It is well documented that the most favourable conformal radiotherapy (CFRT) dose distributions are obtained using three orthogonal fields, as used in the CHHiP trial [24].

Acute bowel toxicity was greater in the FP group, with an approximate doubling in the proportion of patients with RTOG grade 2 + events (FP 50% and IP 21%, respectively) mirrored by a similar increase in PRO moderate or worse symptoms of rectal urgency, distress and overall problems with bowels assessed at week 10. The main toxicity end point in the main CHHiP trial study was grade 2 + RTOG toxicity at 2 years [13]. However, the low level of grade 2 + toxicity observed across the whole trial, as well as in this analysis (only one case each of grade 2 bowel and bladder toxicity), make it an insensitive tool for dissecting differences between FP and IP groups. Although there was no consistent difference in late RTOG toxicity scores, both RMH and LENT/SOM tools showed benefits for IP, with less than half the recorded RMH grade 1 + toxicity (hazard ratio 0.40) and LENT/SOM documented symptoms (hazard ratio 0.48). It is well documented that there are different components to prostate radiotherapy side-effects and proctopathy [25]. The RTOG scale reflects proctitis and bleeding, whereas RMH–LENT/SOM instruments include bowel frequency and looseness. Our previous studies on the impact of different dose levels on bowel symptoms suggest that higher doses in the 60–70 Gy range are associated with bleeding and ‘proctitis’, whereas a moderate dose ‘bath’ of 50–60 Gy is associated with frequency, looseness and sphincter control [26], [27]. In the present study, IP produced both benefits, particularly in the 50–65 Gy dose range for the RMH and LENT/SOM assessments. The favourable PRO in both FP and IP groups underlines the low level of late toxicity seen with both techniques. There were no obvious differences in either acute or late grade 2 + bladder toxicity between FP and IP groups, although the IP group seemed to have a slight increase in grade 1 acute and late side-effects.

The lack of substantial differences in long-term effects between FP and IP methods is in keeping with recent findings from RTOG trial 0126, which showed similar dosimetric advantages of IMRT compared with carefully designed 3DCRT, but with no difference in patient-reported bowel or bladder function [28]. One implication of the impact of the improvement in contemporary radiotherapy treatment is a need to use increasingly sensitive physician- and patient-reported outcome measures to dissect differences between alternative radiotherapy strategies.

## Conclusions

Significant differences were found between the DVHs for FP and IP patients for rectum and bladder. There were some associations between DVH differences and normal tissue effects, which were statistically significant for acute bowel toxicity and for minor levels of toxicity using LENT/SOM and RMH late side-effect bowel subscales favouring IP techniques. Conversely, IP techniques were associated with a small excess of grade 1 bladder side-effects. Both FP and IP planning techniques were associated with low levels of late normal tissue toxicity.

## Conflicts of Interest

D. Dearnaley reports grants from Cancer Research UK during the conduct of the study and personal fees from Takeda, Amgen, Janssen, Astellas, Sandoz and ICR. In addition, D. Dearnaley has a patent issued EP1933709B1. E. Hall reports grants from Cancer Research UK during the conduct of the study and grants from Accuray outside of this study. V. Khoo reports personal fees and non-financial support from Accuray, Astellas and Bayer and non-financial support from Janssen. V. Khoo also reports honoraria for Speakers Bureaus with Accuray, Astellas, Bayer, Ipsen, Janssen, Takeda and Tolmar.
