# Supplementary material for: Forward- and Inverse-Planned Intensity-Modulated Radiotherapy in the CHHiP Trial: A Comparison of Dosimetry and Normal Tissue Toxicity
Source: Clin Oncol (R Coll Radiol). 2019 Sep;31(9):600–10. doi: 10.1016/j.clon.2019.05.002 (PMC6688097; doi:10.1016/j.clon.2019.05.002)
Supplement: Multimedia component 1 [file mmc1.doc]

**Forward and Inverse Planning: APPENDIX**

**Fig S1 CHHiP PTVs and target doses**


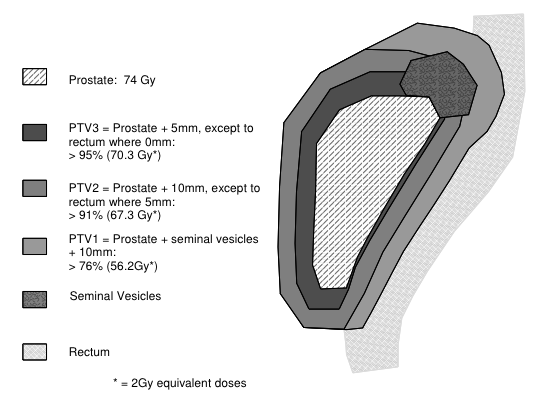


**Fig S2 Effect of matching process illustrated by (A) distribution of rectum and PTV1 volume for all patients (left column) and matched patients (right column) (B) distribution of bladder and PTV2 volume for all patients (left column) and matched patients (right column)**

A – Rectum and PTV1 volumes

B – Bladder and PTV2 volumes

**Table S1 Distribution of matched datasets**

**Distribution of dataset matched for rectum volume and PTV1 (n=156)**

| Rectal volume | PTV1 | | | | | |  |
| --- | --- | --- | --- | --- | --- | --- | --- |
|  | <120cc | 120-160 | 160-200 | 200-240 | 240-280 | >280cc | Total |
| <40cc | 0 | 2 | 7 | 1 | 0 | 0 | 10 |
| 40-60 | 0 | 5 | 19 | 7 | 4 | 1 | 36 |
| 60-80 | 0 | 8 | 6 | 5 | 2 | 1 | 22 |
| 80-100 | 0 | 0 | 3 | 1 | 1 | 1 | 6 |
| 100-120 | 0 | 0 | 0 | 0 | 1 | 1 | 2 |
| >120cc | 0 | 0 | 0 | 2 | 0 | 0 | 2 |
| Total | 0 | 15 | 35 | 16 | 8 | 4 | 78 |

**Distribution of dataset matched for bladder volume and PTV2 (n=172)**

| Bladder volume | PTV2 | | | | | |  |
| --- | --- | --- | --- | --- | --- | --- | --- |
|  | <70cc | 70-100 | 100-130 | 130-160 | 160-190 | >190cc | Total |
| <100cc | 0 | 2 | 1 | 3 | 2 | 0 | 8 |
| 100-175 | 0 | 4 | 9 | 13 | 3 | 1 | 30 |
| 175-250 | 0 | 2 | 12 | 3 | 2 | 1 | 20 |
| 250-325 | 0 | 2 | 6 | 2 | 1 | 0 | 11 |
| 325-400 | 0 | 2 | 1 | 1 | 0 | 0 | 4 |
| >400cc | 0 | 2 | 3 | 4 | 2 | 2 | 13 |
| Total | 0 | 14 | 32 | 26 | 10 | 4 | 86 |

**Distribution of dataset matched for rectum volume, PTV 1 and CHHiP dose schedule (n=106)**

| Rectal volume | PTV1 | | | | | |  |
| --- | --- | --- | --- | --- | --- | --- | --- |
|  | <120cc | 120-160 | 160-200 | 200-240 | 240-280 | >280cc | Total |
| <40cc | 0 | 1 | 6 | 1 | 0 | 0 | 8 |
| 40-60 | 0 | 3 | 18 | 6 | 3 | 1 | 31 |
| 60-80 | 0 | 3 | 2 | 2 | 2 | 0 | 9 |
| 80-100 | 0 | 0 | 2 | 0 | 1 | 0 | 3 |
| 100-120 | 0 | 0 | 0 | 0 | 1 | 0 | 1 |
| >120cc | 0 | 0 | 0 | 1 | 0 | 0 | 1 |
| Total | 0 | 7 | 28 | 10 | 7 | 1 | 53 |

**Distribution of dataset matched for bladder volume, PTV2 and CHHiP dose schedule (n=122)**

| Bladder volume | PTV2 | | | | | |  |
| --- | --- | --- | --- | --- | --- | --- | --- |
|  | <70cc | 70-100 | 100-130 | 130-160 | 160-190 | >190cc | Total |
| <100cc | 0 | 1 | 1 | 0 | 1 | 0 | 3 |
| 100-175 | 0 | 4 | 7 | 12 | 1 | 1 | 25 |
| 175-250 | 0 | 1 | 12 | 1 | 1 | 1 | 16 |
| 250-325 | 0 | 1 | 5 | 1 | 1 | 0 | 8 |
| 325-400 | 0 | 0 | 1 | 0 | 0 | 0 | 1 |
| >400cc | 0 | 1 | 2 | 2 | 2 | 1 | 8 |
| Total | 0 | 8 | 28 | 16 | 6 | 3 | 61 |

**Table S2 Baseline characteristics for rectum-volume matched dataset and bladder-volume matched datasets**

|  | **Rectum volume matched dataset** | | | **Bladder volume matched dataset** | | |
| --- | --- | --- | --- | --- | --- | --- |
|  | **FP** | **IP** | **P value** | **FP** | **IP** | **P value** |
|  | **n=53** | **n=53** |  | **N=61** | **N=61** |  |
|  | **n (%)** | **n (%)** |  | **n (%)** | **n (%)** |  |
| **Age at registration (years)** |  |  |  |  |  |  |
| Median (quartiles) | 68 (64-71) | 67 (63-72) | 0.4 | 68 (64-71) | 66 (62-70) | 0.13 |
| Range | 57-78 | 56-76 |  | 58-79 | 51-78 |  |
| **Weeks from histological confirmation of prostate cancer to randomisation** |  |  |  |  |  |  |
| Median (quartiles) | 26 (18-33) | 22 (19-25) | 0.19 | 22 (15-30) | 22 (19-27) | 0.97 |
| Range | 6-145 | 11-190 |  | 6-143 | 7-93 |  |
| **T stage (clinical assessment)** |  |  |  |  |  |  |
| T1a/T1b/T1c/T1x | 17 (32) | 13 (25) |  | 27 (44) | 17 (28) | 0.1 |
| T2a/T2b/T2c/T2x | 32 (60) | 35 (66) | 0.39 | 29 (48) | 39 (64) |  |
| T3a/T3x | 4 (8) | 5 (9) |  | 5 (8) | 5 (8) |  |
| **Gleason score** |  |  |  |  |  |  |
| ≤4 | 3 (6) | 1 (2) |  | 2 (3) | 1 (2) |  |
| 5-6 | 15 (28) | 13 (25) |  | 13 (21) | 15 (25) |  |
| 7 | 33 (62) | 35 (66) | 0.29 | 44 (72) | 43 (71) | 0.89 |
| 8 | 2 (4) | 4 (8) |  | 2 (3) | 2 (3) |  |
| **PSA (pre-hormone treatment) (ng/ml)** |  |  |  |  |  |  |
| Median  (quartiles) | 14.0  (9.8-17.8) | 11.7  (7.3-17.1) |  | 14.4  (10.4-19.0) | 10.9  (7.3-18.1) |  |
| Mean (SD) | 14.6 (6.4) | 13.1 (6.2) | 0.22 | 15.4 (6.4) | 13.0 (6.2) | 0.04 |
| PSA (ng/ml) |  |  |  |  |  |  |
| 0.0-4.99 | 2 (4) | 1 (2) |  | 1 (2) | 2 (3) |  |
| 5.0-9.99 | 12 (23) | 21 (40) |  | 11 (18) | 26 (43) |  |
| 10.0-19.99 | 29 (55) | 23 (43) |  | 35 (58) | 22 (36) |  |
| 20.0-49.99 | 10 (19) | 8 (15) |  | 14 (23) | 11 (18) |  |
| **Pre-treatment risk group** |  |  |  |  |  |  |
| Low | 0 | 0 | - | 0 | 0 | - |
| Medium | 53 (100) | 53 (100) |  | 61 (100) | 61 (100) |  |
| **Radiotherapy regimen** |  |  |  |  |  |  |
| 74Gy/37f | 19 (36) | 19 (36) |  | 23 (38) | 23 (38) |  |
| 60Gy/20f | 15 (28) | 15 (28) | - | 19 (31) | 19 (31) | - |
| 57Gy/19f | 19 (36) | 19 (36) |  | 19 (31) | 19 (31) |  |
| **Posterior margins modified** Yes | 4 (8) | 0 |  | 6 (10) | 1 (2) |  |
| No | 48 (92) | 52 (100) |  | 54 (90) | 59 (98) | 0.05 |
| Missing | 1 | 1 | 0.04 | 1 | 1 |  |

**Table S3** – Total number of events, hazard ratio and cumulative proportion with events by 2 years for bowel (population A) and bladder toxicity by planning method

|  |  | **Total events** | **HR^1^ (95% CI)** | **P-value^2^** | **2 year cumulative incidence (95% CI)** | **2 year cumulative incidence (95% CI)** |
| --- | --- | --- | --- | --- | --- | --- |
| **BOWEL TOXICITY**  **(rectum volume matched dataset)** | | | | | **FP** | **IP** |
| RTOG | Grade≥1 | 33 | 0.76 (0.38-1.51) | 0.431 | 27.5 (17.3-41.9) | 24.6 (15.1-38.5) |
|  | Grade≥2 | 5 | 0.24 (0.03-2.16) | 0.204 | 5.9 (1.9-17.1) | 1.9 (0.3-12.6) |
|  | Grade≥3 | 2 | 1.18 (0.07-18.9) | 0.906 | - | - |
| RMH | Grade≥1 | 48 | 0.40 (0.21-0.73) | 0.003 | 45.1 (32.7-59.7) | 28.4 (18.2-42.6) |
|  | Grade≥2 | 10 | 0.43 (0.11-1.68) | 0.226 | 7.8 (3.0-19.6) | 3.8 (1.0-14.3) |
|  | Grade≥3 | 4 | 0.35 (0.04-3.34) | 0.360 | 1.9 (0.3-13.1) | 1.9 (0.03-12.7) |
| LENTSOM | Grade≥1 | 54 | 0.48 (0.27-0.84) | 0.010 | 52.9 (40.1-67.0) | 28.3 (18.1-42.5) |
|  | Grade≥2 | 18 | 0.39 (0.14-1.09) | 0.074 | 15.7 (8.2-28.9) | 7.6 (2.9-18.9) |
|  | Grade≥3 | 5 | 0.28 (0.3-2.51) | 0.255 | 3.9 (0.01-14.8) | 1.9 (0.003-12.7) |
| **BLADDER TOXICITY**  **(bladder volume matched dataset)** | | | | |  |  |
| RTOG | Grade≥1 | 20 | 1.58 (0.65-3.87) | 0.316 | 12.2 (6.0-24.0) | 14.9 (8.1-26.7) |
|  | Grade≥2 | 8 | 0.99 (0.25-3.98) | 0.992 | 5.2 (1.7-15.2) | 3.3 (0.1-12.7) |
|  | Grade≥3 | 2 | 1.11 (0.07-18.0) | 0.968 | - | 1.7 (0.2-11.3) |
| RMH | Grade≥1 | 87 | 1.04 (0.68-1.59) | 0.850 | 60.7 (48.5-73.1) | 62.3 (50.4-74.3) |
|  | Grade≥2 | 19 | 0.95 (0.38-2.35) | 0.910 | 10.1 (5.7-21.1) | 8.3 (3.6-18.8) |
|  | Grade≥3 | 8 | 0.35 (0.07-1.75) | 0.203 | 5.1 (1.7-15.1) | 3.3 (0.8-12.7) |
| LENTSOM | Grade≥1 | 89 | 1.58 (1.03-2.43) | 0.037 | 56.3 (44.1-69.3) | 69.6 (57.8-80.7) |
|  | Grade≥2 | 51 | 1.21 (0.69-2.10) | 0.506 | 32.8 (22.3-46.6) | 33.2 (22.9-46.6) |
|  | Grade≥3 | 14 | 0.84 (0.29-2.42) | 0.743 | 6.8 (2.6-17.2) | 5.0 (1.7-14.8) |

^1^ Hazard ratio (HR)<1 favours Inverse planning method

^2^ P-value from Wald test

**Fig S3 Prostate Cancer Index change scores from pre-RT to 24 months**

Positive change scores indicate better QL score at 24 months compared to pre-RT

Negative change scores indicate worse QL score at 24 months compared to pre-RT

NB. Loose stools is scored in the opposite direction from the other bowel habits but the loose stools scoring has been reversed so it is the same direction as all the other bowel items, so all plots can be interpreted in the same way

**A – Bowel habits – change pre-radiotherapy to 24 months (rectum volume matched dataset)**

NB. Overall problem with urinary function is scored in the opposite direction from the other urinary items but the overall urinary problem scoring has been reversed so it is the same direction as all the other urinary items, so all plots can be interpreted in the same way

**B – Urinary function change from pre-radiotherapy to 24 months (bladder volume matched dataset)**
